# Nano‐Enhanced Graphite/Phase Change Material/Graphene Composite for Sustainable and Efficient Passive Thermal Management

**DOI:** 10.1002/advs.202402190

**Published:** 2024-08-09

**Authors:** Ji‐Xiang Wang, Yufeng Mao, Nenad Miljkovic

**Affiliations:** ^1^ Institute of Optics and Electronics Chinese Academy of Sciences Chengdu 610209 P. R. China; ^2^ Hebei Key Laboratory of Man‐machine Environmental Thermal Control Technology and Equipment Hebei Vocational University of Technology and Engineering Hebei 054000 P. R. China; ^3^ Department of Mechanical Engineering The Hong Kong Polytechnic University Hung Hom Kowloon Hong Kong SAR P. R. China; ^4^ College of Electrical Energy and Power Engineering Yangzhou University Yangzhou 225009 P. R. China; ^5^ Department of Mechanical Science and Engineering University of Illinois Urbana‐Champaign Urbana IL 61801 USA; ^6^ Department of Electrical and Computer Engineering University of Illinois at Urbana‐Champaign Urbana IL 61801 USA; ^7^ Materials Research Laboratory University of Illinois Urbana IL 61801 USA; ^8^ International Institute for Carbon‐Neutral Energy Research (WPI‐I2CNER) Kyushu University 744 Motooka, Nishi‐ku Fukuoka 819‐0395 Japan; ^9^ Institute for Sustainability, Energy and Environment (iSEE) University of Illinois Urbana IL 61801 USA

**Keywords:** battery safety, graphene, passive thermal management, phase change composite, thermal radiation

## Abstract

Passive battery thermal management systems (BTMSs) are critical for mitigation of battery thermal runaway (TR). Phase change materials (PCMs) have shown promise for mitigating transient thermal challenges. Fluid leakage and low effective thermal conductivity limit PCM adoption. Furthermore, the thermal capacitance of PCMs diminishes as their latent load is exhausted, creating an unsustainable cooling effect that is transitory. Here, an expanded graphite/PCM/graphene composite that solves these challenges is proposed. The expanded graphite/PCM phase change composite eliminates leakage and increases effective thermal conductivity while the graphene coating enables radiative cooling for PCM regeneration. The composite demonstrates excellent thermal performance in a real BTMS and shows a 26% decrease in temperature when compared to conventional BTMS materials. The composite exhibits thermal control performance comparable with active cooling, resulting in reduced cost and increased simplicity. In addition to BTMSs, this material is anticipated to have application in a plethora of engineered systems requiring stringent thermal management.

## Graphical abstract

A scalable, durable, lightweight, compact, leak‐free graphite/phase change material/graphene composite with high effective thermal conductivity is developed for battery thermal management applications. The composite efficiently absorbs battery heat and releases it to the environment using its intrinsically high thermal conductivity and high surface emissivity. This composite material enables passive cooling for potential application in aerospace, building, and mobile applications.

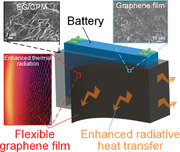

## 1 Introduction

Electrification is a global trend that seeks to lower the dependence on fossil fuel energy and mitigate the adverse effects of climate change.^[^ ^1^ ^]^ The transition to electrochemical engines requires improved batteries.^[^ ^2^ ^]^ Due to their high energy density, long lifetime, and low self‐discharge rates,^[^ ^3^ ^]^ lithium‐ion batteries (LIBs) offer high levels of electrochemical power and energy with advantages over other battery materials. However, thermal runaway (TR)^[^ ^4^, ^5^ ^]^ of LIBs is widely considered an issue.^[^ ^6^, ^7^ ^]^ TR is a catastrophic battery failure mode which causes fires,^[^ ^8^ ^]^ threatens lives, and causes destruction of systems and sub‐systems. Therefore, understanding TR mechanisms, suppressing TR, and enhancing battery safety is a top priority.^[^ ^9^ ^]^ TR commonly takes place when the LIB temperature reaches above 90 °C. At these elevated temperatures, undesired side reactions,^[^ ^10^ ^]^ rapid anode degradation,^[^ ^11^ ^]^ and material decomposition^[^ ^12^ ^]^ inside the battery occur. These high operating temperatures can occur when the battery undergoes rapid charging or discharging.^[^ ^13^ ^]^ As a result, advanced battery thermal management systems (BTMSs), which can maintain the battery temperature within an optimal temperature range (15–60 °C^[^ ^14^ ^]^), become a significant element of LIB design^[^ ^15^ ^]^ and represent a very active research topic within the science and engineering communities.^[^ ^16^, ^17^ ^]^

**Figure 1:**
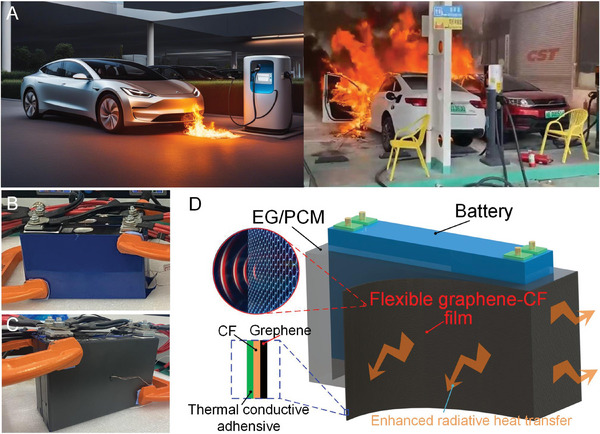
Electric vehicle battery challenges and proposed solution. A) Photographs showing TR‐induced fires on charging electric vehicles. The right‐side photographs obtained from NetEase News with permission. B) Battery without BTMS (Group A). C) Battery directly coated with a graphene layer (Group B). D) Schematics of the EG/PCM/graphene composite for passive BTMS thermal management (Group D). Group C is defined as a battery pack having the EG/PCM composite without the graphene coating. For Group C, these battery pack was inserted in the EG/PCM tightly to ensure an efficient heat transfer from the battery to the EG/PCM. A graphene film was attached to the outer surface of the EG/PCM to achieve an enhanced radiative heat transfer. The graphene film is a three‐layer structure where the graphene (outer layer) was coated upon a copper foil (CF) (intermediate layer). The other side of the CF was applied to the thermal conductive adhesive to form the bottom layer which is attached tightly to the outer surface of the EG/PCM.

Until now, BTMS challenges associated with TR remain unsolved and must be mitigated via rigorous monitoring and performance‐limiting safety precautions. The primary challenge to BTMS development is the trade‐off between energy consumption, weight/volume, and cooling capacity.^[^ ^18^ ^]^ An active BTMS with a heavier weight and larger volume commonly has a relatively large cooling capacity.^[^ ^19^ ^]^ However, these larger systems consume more energy, reducing electric vehicle range. Worse yet, active liquid cooling schemes can lose power and thus, cooling ability while the vehicle is unpowered or parked and charging. This loss of power limits suppression of TR from accumulated heat in the unpowered vehicle. This is the main reason why TR‐induced fires are the most common on vehicles undergoing charging^[^ ^20^ ^]^ (**Figure** **1**A). Therefore, a passive BTMS, which can operate in the unpowered and charging state,^[^ ^21^ ^]^ is preferred from the standpoint of electric vehicle safety.

Among all passive thermal control strategies, phase change materials (PCMs) are one of the most promising.^[^ ^22^, ^23^ ^]^ The PCM works by using a solid‐liquid phase transition,^[^ ^24^, ^25^ ^]^ thus enabling the absorption of heat at a relatively constant temperature.^[^ ^26^ ^]^ Hence, high density cooling can be achieved at a regulated temperature. Although promising, PCMs have not been applied extensively due to their relatively low intrinsic thermal conductivity (0.1−0.2 W m^−1^ K^−1^ for organic PCMs)^[^ ^27^ ^]^ as well as liquid PCM leakage stemming from high volumetric expansion during melting.^[^ ^28^, ^29^ ^]^

Efforts have been made toward solving these two drawbacks. A PCM integrated with a high thermally conductive and porous skeleton, termed phase change composite (PCC), is regarded as a promising approach. Many nano‐ and microscale materials such as metal foams,^[^ ^30^, ^31^ ^]^ ceramics,^[^ ^32^ ^]^ inorganic compounds,^[^ ^33^, ^34^ ^]^ metal‐organic frameworks,^[^ ^35^ ^]^ and graphene‐based materials^[^ ^36^ ^]^ have been proposed to achieve form‐stable, thermally conductive, and leakage‐free PCCs. In particular, expanded graphite (EG)^[^ ^38^, ^39^ ^]^ commonly has multiple highly porous layers,^[^ ^37^, ^40^ ^]^ which support PCM skeleton formation.^[^ ^41^, ^42^ ^]^

Pure PCM passive thermal management suffers from insufficient and unsustainable cooling^[^ ^43^, ^44^ ^]^ due to limited heat release capability.^[^ ^45^ ^]^ Although the match between the heat load and the PCM total latent heat matters,^[^ ^46^ ^]^ the latent heat of the PCM will be exhausted without subsequent cooling, and the remaining liquid‐phase PCM is a poor thermal management material. Hence, PCM‐based BTMS should be integrated with air, liquid, and/or thermoelectric cooling^[^ ^47^, ^48^, ^49^ ^]^ to efficiently dissipate the heat accumulated inside the PCM.^[^ ^50^ ^]^ This in turn transforms the liquid‐phase PCM back to the solid‐phase and makes the method sustainable over many drive cycles. However, onboard active cooling schemes are mainly available for driving electric vehicles. The cooling demand may exceed the cooling supply for an unpowered car. Therefore, exploring completely passive PCM‐based BTMSs is a potential solution to enable greater electric vehicle safety. Radiative cooling,^[^ ^51^ ^]^ which passively dissipates heat away via electromagnetic waves enables sustainable cooling.^[^ ^52^ ^]^ Thus, seeking cutting‐edge radiation materials that can result in high compatibility with PCCs is imperative for future BTMS development. It has been reported that nanosheet graphene is a promising thermal emitter,^[^ ^53^, ^54^ ^]^ indicating that nanostructured graphene potentially can be co‐implemented with form‐stable PCCs.

Here, we design and fabricate an EG/PCM/graphene composite that functions as a passive BTMS (Figure 1D). The EG/PCM works as a PCC that is wrapped tightly around a battery pack where the generated heat can be transferred from the pack to the PCC. The PCM enables thermal control while the EG inside the PCC enables high effective thermal conductivity and leakage minimization. Nanostructured graphene was coated on one side of a flexible copper foil (CF). The other side of the copper foil was coated with a thermally conductive adhesive so that the graphene‐CF can be tightly attached to the outer surface of the PCC to form the EG/PCM/graphene composite. The graphene outer surface can efficiently dissipate heat generated inside the PCC via thermal radiation. Battery charging–discharging experiments show that the proposed composite reduces the battery temperature with zero energy consumption when compared to other approaches. Our work rationally combines an optimized PCC with radiative cooling materials, creating a passive BTMS. More importantly, we demonstrate scalable manufacturing of a durable high‐performing thermal control material, vital for promoting safer electrification and decarbonization of society.

## 2 Results

### 2.1 Expanded Graphene and Phase Change Material Characterization

**Figure 2:**
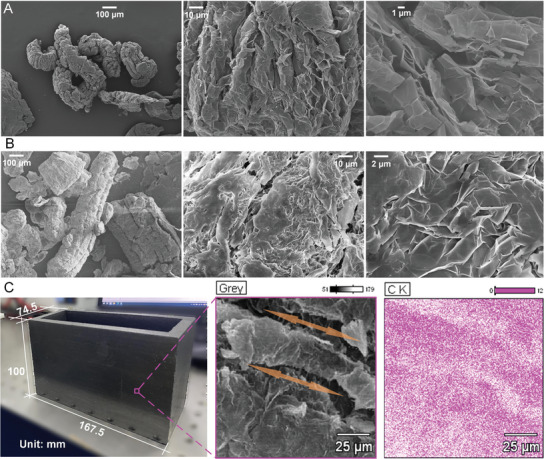
Characterizations of the EG and EG/PCM composite. A) Scanning electron microscopy (SEM) images of the utilized EG. B) SEM images of the EG/PCM particles. C) Photograph of the compressed EG/PCM composite for BTMS and its SEM characterization (middle panel) with element mapping (right panel).

The utilized EG was thermally exfoliated and unfolded from natural graphite flake particles through a microwave heat treatment (**Figure** **2**A). A fine nanocellular structure was observed on the flakes. The nanoscale cavities within the cellular‐structure provide sufficient space for PCM loading and capillary force generation for liquid‐PCM leakage prevention. We used organic PCM (paraffin wax) to synthesize the EG/PCM PCC (Figure 2B). After PCM impregnation, the PCC demonstrates almost identical morphology as the EG. These structures were then coated with PCM particles and heated to a liquid‐state to form a PCM layer on top of the EG. Scanning electron microscopy (SEM) images in Figure 2B show a uniformly distributed PCM within the EG. The composite was complete after pressure shaping of the EG/PCM using a mold whose length × width × height is 167 ± 0.5 mm × 74 ± 0.5 mm × 100 ± 0.5 mm (Figure 2C). The PCC wall thickness was 10 ± 0.5 mm. The resultant compact PCC has a mass of 344 ± 1 g and a density of 775 ± 5 kg m^−3^. Detailed fabrication procedures for the PCC are described in Section S1 (Supporting Information). A SEM image of the composite after pressure forming is presented in Figure 2C, where an orderly layer‐by‐layer structure with aligned and pressurized EG/PCM frameworks is shown. The SEM image shows the presence of a fully dense material without obvious cellular‐like structures after pressure‐shaping. Carbon elemental mapping of the pressurized EG/PCM by an energy dispersive spectrometer is also shown in Figure 2C. The thermal conductivity of the EG/PCM was measured to be 16.2 ± 2 W m^−1^ K^−1^, which is higher than that of the pristine paraffin (≈0.2 W m^−1^ K^−1^). See Section S2 (Supporting Information) for thermal conductivity measurement device details and methodology.

Differential scanning calorimetric (DSC) curves of the pristine paraffin wax and EG/PCM are plotted in Section S3 (Supporting Information). The melting point of the resultant EG/PCM PCC was determined to be 48.3 ± 2 °C with a latent heat of 216.1 ± 7.0 kJ kg^−1^. Therefore, the thermal storage capacity is calculated to be 74.3 ± 1.0 kJ for the prototype. Please refer to Table S1 for detailed parameters of both the pristine paraffin wax and PCC prototype. The mass fraction of the paraffin wax inside the PCC, defined by Equation (1), was calculated to be 80.8 ± 0.5%. This value agrees with initial design estimates used to help prevent paraffin leakage.^[^ ^55^ ^]^

(1) $$\gamma =\left(\Delta H_{m,PCC}+\Delta H_{s,PCC}\right)/\left(\Delta H_{m,p}+\Delta H_{s,p}\right)\times 100\%$$

where Δ*H*_*m*,*p*_ and Δ*H*_*s*,*p*_ are the melting and solidification latent heats of the pristine paraffin wax. Here, Δ*H*_*m*,*PCC*_ and Δ*H*_*s*,*PCC*_ are the melting and solidification latent heats of the EG/PCM materials. Detailed values of the parameters used in Equation (1) are listed in Table S1 (Supporting Information).

### 2.2 Graphene Coating Characterization

**Figure 3:**
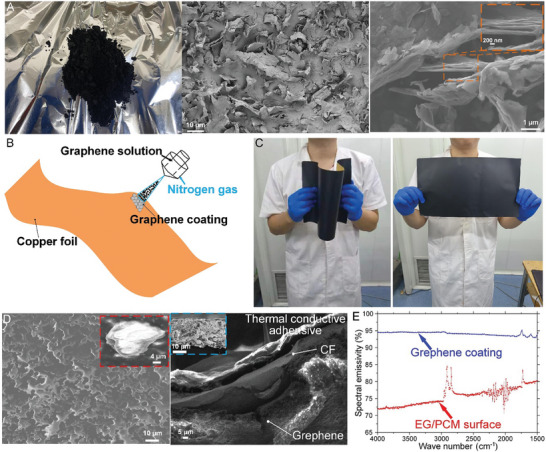
Characterization of graphene and graphene coatings. A) Photograph (left) and SEM images (middle and right) of the graphene particles. The inset demarked by the orange dashed lines indicates that the thickness of the nanosheet graphene is ≈10 nm. B) Schematic of the graphene coating process on a flexible CF sheet (orange color). C) Demonstration of the flexibility of the graphene coated CF. D) SEM images of the graphene coated CF with the adhesive on the other side of the CF. The insert marked by red shows a magnified view of a nanosheet graphene. The inset demarked by the blue dashed lines represents the cross‐sectional view of the graphene coating layer on CF. The thickness of the graphene coating is ≈20 µm. E) Spectral emissivity comparison between the EG/PCM composite outer surface and the graphene coating in the mid‐infrared (MIR) region (4000–1500 cm^−1^).

**Figure** **3**A shows a photograph and SEM images of the utilized chemical vapor deposited (CVD)‐based graphene particles. The low thickness/length ratio of the nanosheet graphene indicates a high thermal radiation capability. The graphene coating process on the 50 µm thick CF is demonstrated in Figure 3B through an ultrasonic spray technology where the prepared graphene solution (see Section S4 of the Supporting Information for the solution preparation procedure) is sprayed on the CF with a stream of high‐pressure nitrogen gas (see Section S5 of the Supporting Information for the detailed spray coating process). The high flexibility of the graphene‐coated CF is shown in Figure 3C.

SEM images of the graphene coating deposited on the CF are displayed in Figure 3D. The images show that the nanosheet graphene plate, responsible for enhancing thermal radiation, is well adhered. A sectional view of the CF with one side coated with graphene and the other side with thermally conductive adhesive is presented in the right panel of Figure 3D where an obvious three‐layer structure is observed. To quantitatively characterize the coatings critical function, Figure 3E compares the spectral emissivity of both the graphene coating and the PCC outer surface in the mid‐infrared region (MIR). The graphene coating, which is added to help prevent battery TR, needs to be operated at temperatures well below 100 °C. At these temperatures, the thermal radiation window resides well within the MIR spectrum as displayed in Figure 3E. A high wavelength‐averaged spectral emissivity of 0.946 (as calculated by Equation 2)^[^ ^57^ ^]^ for the graphene coating was obtained in the MIR region. For the EG/PCM PCC outer surface, it was only 0.76. Thus, an emissivity enhancement of 25.1% in thermal radiation capability was attained.

(2) $$\bar{\varepsilon \left(T\right)}=\frac{\int_{1500\;cm^{-1}}^{4000\;cm^{-1}}\varepsilon \left(\sigma \right)I_{b}\left(\sigma ,T\right)d\sigma }{\int_{1500\;cm^{-1}}^{4000\;cm^{-1}}I_{b}\left(\sigma ,T\right)d\sigma }$$

where ε(σ) is the emissivity at wave number σ and temperature *T*. Here, *I_b_* (σ,*T*) represents blackbody radiation at wave number σ and temperature *T*.

Further standard characterization^[^ ^58^ ^]^ of the graphene coating including Raman spectra, elemental mapping, X‐ray fluorescence (XRF) and X‐ray photoelectron spectroscopy (XPS)^[^ ^59^, ^60^ ^]^ results are presented in Section S6 (Supporting Information).

### 2.3 Thermal Experiments

Thermogravimetric analyses of both the EG/PCM particles and graphene coating (see Section S7, Supporting Information) illustrate high thermal stability of the composite below the battery TR temperature. Therefore, thermal experiments using the EG/PCM/graphene composite were conducted. Four groups (A, B, C, and D) of batteries with BTMSs were adopted in the battery discharge‐charge experiments (see Experimental Section for details). Two battery cells (see Section S8, Supporting Information for detailed parameters of these battery cells) without any BTMS were utilized as a set of reference experiments, demarked as Group A and shown in Figure 1B. The original blue surface of the battery cell was characterized and is described in Section S9 (Supporting Information). Group B consists of the same two battery cells having a direct graphene coating. Due to the coating, the cell color changes from blue to black, as shown in Figure 1C. Group C consists of the same two batteries integrated with the EG/PCM PCC (see Figure S9, Supporting Information). Group D, in which these two battery cells were wrapped with EG/PCM/graphene composite, represents the focus of this paper and the prototype studied herein.

**Figure 4:**
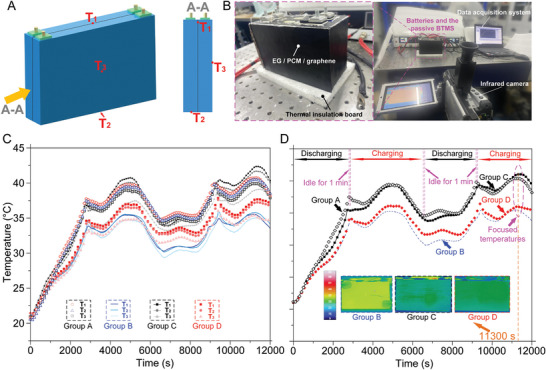
Battery thermal experiment relations and the experimental data demonstration. A) Schematics of the temperature detection locations upon the batteries’ surface. B) Digital image of the battery thermal management of the Group D. C) Three temperature curves for each group at the environmental temperature of 20 °C and charging/discharging current of 1.25 C. D) average temperature curve for each group at the same experimental condition as (c). The inserted images are the infrared images of three groups (B, C, and D) at the time of 11 300 s.

To study the thermal behavior of the batteries, three thermocouples (*T*_1_, *T*_2_, and *T*_3_) were attached to the outer surface of the batteries as shown in **Figure** **4**A. For a detailed description of the experimental sensors and uncertainty, see the Materials and devices section. Here, *T*_1_ represents the temperature at the interface of these two battery cells, *T*_2_ represents the temperature at the bottom surface of one of the batteries, and *T*_3_ represents the temperature at the side wall of the same battery. Figure 4B shows a photograph of the thermal experimental setup highlighting Group D experiments where the battery cells are inserted into the EG/PCM/graphene composite.

Figure 4C shows the temperature response curves for the four groups of batteries at the 1.25C charging/discharging current and 20 °C environmental temperature. Generally, *T*_1_ is always the highest when compared to the other two temperature points because it is in the middle of two battery surfaces. The temperature *T*_3_ is the lowest one as the ambient air can easily cool down the side of the battery. For groups of A and B, the difference among these three temperatures is small, indicating a relatively even temperature distribution while for groups of C and D, the temperature differences between *T*_3_ and the other two temperatures (*T*_1_ and *T*_2_) are usually large. Such large temperature divergence is because *T*_3_ is tightly contacted with the EG/PCM PCC, which can significantly suppress the temperature. Although the temperature in this operating condition can barely reach the melting point of the PCM, the specific heat alone of the PCC can decrease the temperature. It is expected that such temperature differences become larger when temperatures increase to or above the PCM melting point. To simplify the analysis, the average temperature calculated by (*T*_1_ + *T*_2_ + *T*_3_)/3 is utilized to represent the overall temperature of the batteries, as presented in Figure 4D. For each temperature curve, there are four temperature peaks representing the maximum temperature in each discharging or charging state. The last temperature peak represents the maximum temperature during the last charging process. This peak is always relatively high (representing the most dangerous time) when compared to other three temperature peaks. Therefore, the temperature during this third peak for each group is the focus and is compared between the groups as a critical parameter. The temperatures for Groups A and C are the highest while for Group B it is the lowest (with a temperature difference of 5.2 °C), suggesting that the graphene directly coated on the battery surface is the best thermal management scheme for this condition. The inferiority of Group C comes from the relatively low temperature where the latent heat of the PCM is hardly utilized. The reason why the temperature of Group D is larger than that of Group B can be attributed to the Stefan‐Boltzmann law, described as Equation (3). Here, *T_ra_* of Group D is lower than that of Group B, validated by the inset images in Figure 4D, because the EG/PCM functions as a layer of thermal resistance, which results in a decreased *Q_ra_* for Group D and hence a higher temperature.

(3) $$Q_{ra}=\varepsilon \kappa A\left(T_{ra}^{4}-T_{e}^{4}\right)$$

where ε is the spectrally averaged emissivity of the radiation surface, κ is the Stefan‐Boltzmann constant, *A* is the area of the radiation surface, *T_ra_* is the temperature of the radiation surface, *T_e_* is the environment temperature, and *Q_ra_* is the total heat transferred via radiation.

**Figure 5:**
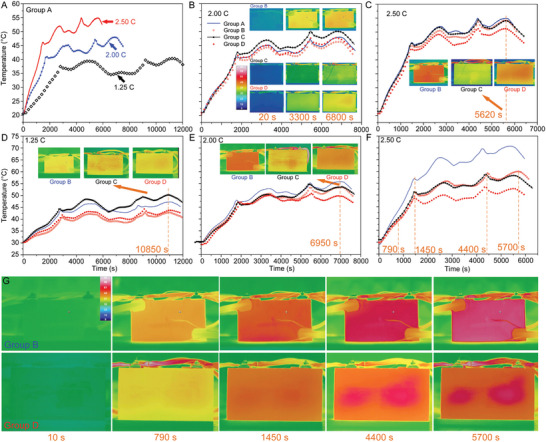
Battery thermal response at various operating conditions. A) Temperature curve for Group A at different discharging/charging current. B−C) Temperature curve for each group at the current of B) 2.00 C and C) 2.50 C. The environment temperature for A), B), and C) is 20 °C. D−F) Temperature tendencies at the environmental temperature of 30 °C for each group at the current of D) 1.25 C, E) 2.00 C, and F) 2.50 C. Inserted images in A−F) represent the infrared images at certain moments. G) Infrared images for Groups B and D at five typical moments in the identical operating condition as F).

**Figure** **5** presents the experimental results for a wider set of operating conditions. Figure 5A plots the temperature response of Group A at different discharging/charging currents at an environmental temperature of 20 °C. At higher currents, the test time becomes shorter and the temperatures higher, indicating the passive BTMS is indispensable for high current charging. Figure 5B shows the temperature trends of these four groups at a charging rate of 2C. Unexpectedly, it shows that the temperature of Group C is always the largest (maximum temperature of ≈50 °C), indicating that the EG/PCM composite alone deteriorates the heat dissipation rate as the latent heat of the PCM has not been utilized due to the low temperature (still below 50 °C). The temperatures of Groups A and D are very similar, indicating the non‐necessity of the BTMS, which is caused by the increased natural convection heat dissipation owing to the enlarged temperature difference between the battery and environment. However, the added EG/PCM prevent such convection, causing unnecessary thermal resistance. The temperature of Group B is still the smallest due to the combined enhancement in natural convection and thermal radiation.

As the current increases to 2.5C (Figure 5C), the temperature of Group A become marginally larger than Group C as the thermal buffering effect of the PCM surpasses the cooling effect of natural convection. The temperature of Group D was the lowest in this condition where the temperature difference between A and D was ≈4 °C, proving that the EG/PCM/graphene composite has advantages when compared to the other designs. We can observe the change brought by the graphene coating from the inset images in Figure 5C. With the layer of graphene coating in Group D, the absorbed heat inside the PCC can be transferred out of the battery continuously with a surface of higher temperature when compared to Group C. Although the radiation surface temperature of Group B is even higher than that of Group D, the enhanced radiation cannot surpass the synergistic effects brought forth by both radiation and the PCM latent heat.

Experimental results of various currents at an environmental temperature of 30 °C are displayed in Figure 5D–G. Similar to the results for the 1.25C charging at 20 °C environmental temperature, the temperature of Group C remains the highest (just below the melting point of the PCM), even larger than Group A which has no BTMS. This indicates that the generated heat is low enough so that that natural convection can dissipate this heat effectively. In this situation, a direct coating on the battery surface (Group B) is preferred, showing a 14.6% temperature decrease when compared to Group C. The temperature of Group D is slightly larger when compared to Group B, indicating the benefit brought by the graphene coating. This benefit is attributed to the fourth‐power dependence of radiation on surface temperature. The inset infrared images of Figure 5D indicate that the radiation states for Groups B and D are comparable where the heat absorbed by the EG/PCM is sufficiently removed by the graphene coating without any heat accumulation. However, without the graphene coating, the absorbed heat cannot be dissipated effectively, causing an increase in the surface temperature of the Group C prototype. As expected, the temperature of Group D becomes the smallest at the 2C charging rate (shown in Figure 5E) where the maximum temperature just reaches 50 °C, meaning the latent heat has not been exploited yet. At the same condition, the maximum temperature of Group A reaches ≈56.1 °C (the largest). The temperatures for Groups B and C are similar where the single effect from the phase change and thermal radiation is comparable.

As the current increases to 2.5C, the superiority of the phase change and thermal radiation is manifested. The maximum temperature of Group D is as low as 52.5 °C while Group A is 71.1 °C. The 25.9% temperature decrease is obtained by employing the EG/PCM/graphene composite. At 2.5C, Group C (without the graphene coating) reached a peak temperature of 58 °C, exceeding the paraffin melting point of 54.1 °C. This indicates that the paraffin has fully transitioned to a liquid state, thereby ceasing its latent cooling efficacy. Under the identical condition, Group D manages to keep the temperature below 52.5 °C, indicating that the paraffin latent heat capacity had not been entirely depleted. The retained latent heat is due to the enhanced radiative cooling facilitated by the graphene. The temperature of Group C is clearly smaller than Group B for the first time, indicating that the generated heat from the battery is so large that the thermal radiation can no longer compete with phase change heat transfer. The infrared images shown in Figure 5G shows that the radiation surface temperature of Group B is always larger than that of Group D, indicating an enhanced thermal radiation from Group B. However, this superiority of Group B can be overshadowed by the large latent heat of the paraffin. The infrared movie, designated as Movie S1 (Supporting Information), provides a comprehensive visual record of the experimental process on Group D (charging/discharging current: 2.50 C; environmental temperature: 30 °C). Movie S1 (Supporting Information) also shows that there is no liquid‐phase PCM leakage throughout the entire experiment.

**Figure 6:**
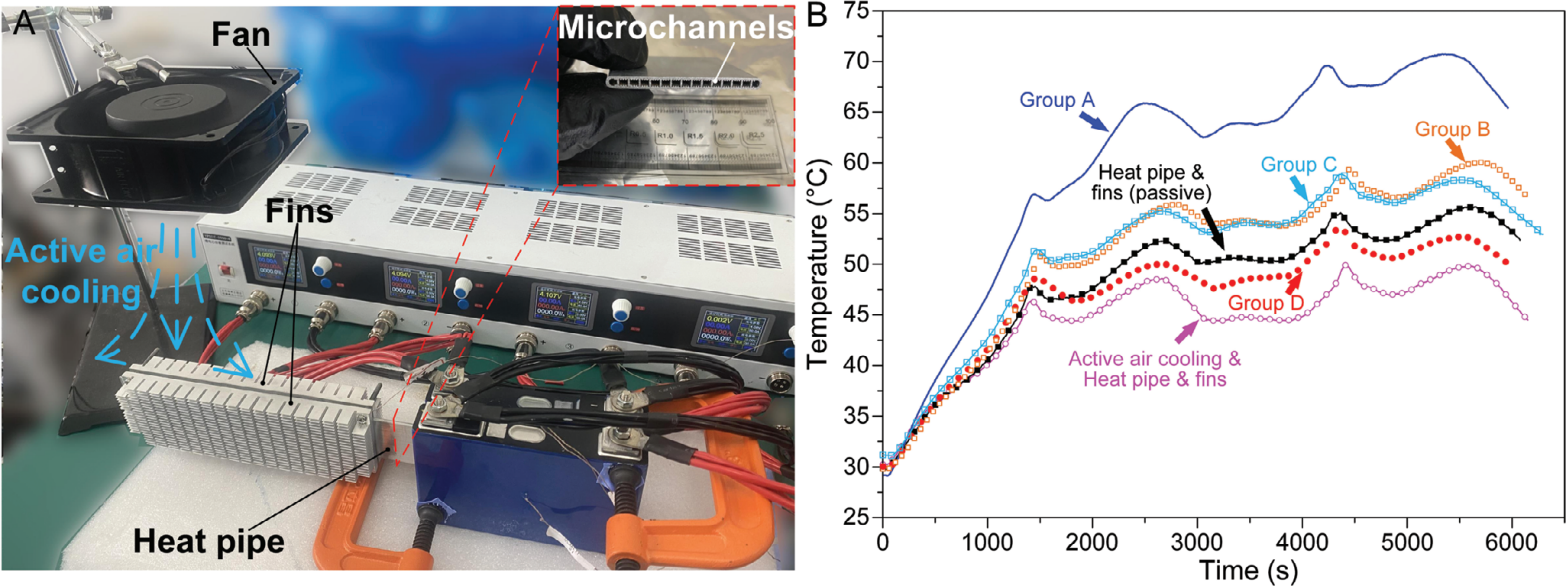
Comparison between the active and EG/PCM/graphene passive BTMSs. A) Digital view of the active BTMSs using a grooved heat pipe, fins, and active air cooling. The insert in (A) shows a cross sectional view of the microchannel heat pipe structure. B) Thermal comparison among the various BTMS approaches. The temperature data of the Group A, B, and C experiments is plotted as a reference. Temperature uncertainty is ±0.1 °C and is not potted for clarity.

To further test the performance of the developed PCC material, a BTMS using a grooved heat pipe and fins, as shown in **Figure** **6**, was tested. As shown in Figure 6A, a side of a commercial aluminum grooved heat pipe was inserted between the battery cells. The inserted region was the evaporator half of the heat pips, while the exposed region with fins represents the condenser half. Above the fins, a fan was placed to create a downward forced air stream where an active air cooling (air velocity: ≈0.45 ms^−1^) can be obtained (see Section S11, Supporting Information for detailed description of the active cooling scheme). Both passive (without fan) and active (with fan) heat pipe cooling experiments at 2.50 C and 30 °C environment temperature were conducted as displayed in Figure 6B. The temperatures of Group D are lower than that of the passive heat pipe approach where the temperature of Group D decreases by 3.1 °C. Therefore, the EG/PCM/graphene composite performs better compared with the heat pipe approach under the passive cooling condition. Furthermore, this enhancement becomes even more advantageous upon acknowledging that our proposed system, weighing in at 344 g, exhibits a 43.6% reduction in mass relative to the heat pipe cooling scheme, which was measured to weigh 610 g as detailed in Section S11 (Supporting Information). When compared with the active air cooling and heat pipe scheme, the temperature of Group D is s larger than, but comparable with that of the active cooling scheme (49.9 °C).

The successful implementation of the proposed EG/PCM/graphene is contingent not only on its superior performance but also on its high durability and low flammability. Please refer to Section S12 (Supporting Information) for details of the successful durability test conducted on the composite. Section S13 and Video S2 (Supporting Information) detail the flammability test of the composite. The composite did not ignite under the flame of an alcohol burner, demonstrating good flame retardancy and applicability in battery systems.^[^ ^76^, ^77^ ^]^

## 3 Discussion

In this paper, we design and fabricate an EG/paraffin/graphene composite that functions as a passive BTMS. The EG/paraffin works as a common phase change composite (PCC). Additionally, nanostructured graphene was coated on the outer surface of the EG/paraffin volume to act as a passive thermal emitter to the external ambient. The battery heat absorbed by the EG/paraffin can be efficiently dissipated into environment via the graphene‐induced thermal radiation. This synergistic combination represents the uniqueness of our work. Although our composite did not always have the best performance when compared to other BTMS approaches, poorer performance occurred for low discharge/charge current conditions and low environment temperatures, where batteries have a low probability of entering the TR state. The developed EG/PCM/graphene composite functions the best at high discharge/charge currents and in adverse heat transfer environments conducive to TR.

Although we did not conduct tests at battery temperatures near TR due to safety concerns and device restrictions, the observed trends indicate that the hybrid material functions the best near the TR condition where both phase change and thermal radiation effects can be realized efficiently, thus delaying the occurrence of TR. For example, the non‐linear 4th power of thermal radiation $\left(∼(T_{ra}^{4}-T_{e}^{4})\right)$ ensures preference for higher battery temperatures.

When compared with other battery cooling strategies such as heat pipes, heat pumps, and immersion cooling, the proposed material offers advantages in significant weight and volume reduction while maintaining a comparable cooling performance. Although heat pipes are a passive technology, they cannot provide sustainable cooling without active cooling. For the identical passive battery thermal management scheme shown in Figure 6B, the average battery temperature using the heat pipe during the entire experiment is 48.7 °C, ≈2 °C larger than the EG/PCM/graphene composite. The maximum battery temperature using the heat pipe is ≈3.1 °C larger than the EG/PCM/graphene. However, the volume occupied by the heat pipe with active cooling is more than twice that of the composite prototype. Its total mass of 610.2 g is much heavier than the proposed PCC design. The results demonstrate the advantage of the EG/PCM/graphene in terms of temperature control, volumetric power density, and gravimetric power density, translating into higher battery performance, longer battery life (an elevated temperature accelerates battery aging^[^ ^58^ ^]^), and longer vehicle range. For active heat pump solutions,^[^ ^62^ ^]^ many other components are needed. Heat pumps also demand additional technologies such as control algorithms^[^ ^63^ ^]^ and defrosting techniques^[^ ^64^ ^]^ to maintain sustainable operation, significantly increasing maintenance costs and range‐anxiety. Immersion cooling is suitable for high heat flux dissipation. However, it needs large amounts of coolant to immerse the entire battery, which makes the BTMS heavier than any other option, significantly decreasing the vehicle's range. Furthermore, the heated coolant needs to be cooled using additional active cooling methods. For passive BTMS, the PCC represents a cost‐effective material which provides sustainable cooling for an unpowered car during charging. Its thermal performance is comparable to that of active cooling schemes, indicating sustainability and sufficient cooling capability despite being passive. Furthermore, the developed radiative cooling materials can also be applied to other cooling schemes such as fin heat sinks in heat pipes and heat removal components which dissipate heat from the system to outer space in heat pump and immersion cooling systems.

Our experiments demonstrate that the EG/PCM/graphene composite has high scalability and compatibility with battery systems. Such materials can be applied as a battery packaging strategy to achieve the purpose of passive thermal management, or act as a supplementary battery cooling method for unpowered vehicles. Aside from its application in battery systems, the proposed composite material can also serve as a sustainable and energy‐saving cooling medium for computer chips,^[^ ^65^, ^66^ ^]^ laser systems,^[^ ^67^, ^68^ ^]^ high‐power energy transmission devices,^[^ ^69^, ^70^ ^]^ electromagnetic equipment,^[^ ^71^, ^72^ ^]^ highly integrated circuits,^[^ ^73^, ^74^ ^]^ and building thermal comfort control.^[^ ^75^ ^]^

## 4 Conclusions

In summary, we developed a scalable, durable, lightweight, compact, leak‐free EG/PCM/graphene PCC with high effective thermal conductivity as a BTMS. The PCC has an energy storage capacity of ≈74.3 kJ and an effective thermal conductivity of 16.2 W m^−1^ K^−1^. The graphene coating has a high averaged emissivity of 0.946 in the mid‐infrared region. The PCC was utilized as a passive BTMS proof of concept demonstration. The generated heat from batteries was shown to be efficiently absorbed by the PCC with limited temperature elevation owing to the PCM phase transition. Our results demonstrate that the accumulated heat inside the PCC can be released into the environment by two mechanisms: the high PCC effective thermal conductivity and the high thermal emissivity of the graphene layer. We show that during passive battery thermal management experiments, the battery temperature can be reduced from 71 °C (without thermal management) to 52.5 °C using the developed PCC. Further comparison with active and passive heat pipe BTMS demonstrates high cost‐effectiveness of the PCC. In addition to BTMS, the proposed EG/PCM/graphene composite is expected to have wider application in aerospace, data center, building, and mobile applications, where significant value can be derived from carbon neutral thermal management techniques.

## 5 Experimental Section

### Materials and Instruments

Absolute ethyl alcohol (model: E111989), Di (propylene glycol) methyl ether (model: D108833), 1‐Methyl‐2‐pyrrolidinone (model: M103246) were procured from Aladdin Bio‐Chem Technology Corporation (Shanghai). Waterborne synthetic polyurethane (model: Leasys 1258) was procured from the Wanhua Chemical Group Corporation. The pristine paraffin wax (model: PCM‐A‐48H) was procured from Zhongjia New Material Technology Ltd. The utilized EG (80 mesh) was procured from Hengrunda Ltd. (Qingdao). The CVD‐based nanosheet graphene (model: WJSG1410) was procured from Vigon Technology Corporation. The thermally conductive adhesive (model: k‐5207) was procured from Kafuter Thailand. The copper foil was procured from Jiayuan Technology Ltd. The heat pipe (model: HP01) was procured from Xuzhou Reer Electronic Technology Ltd. and the two fin types (model: CZ0001) were donated by Zhaodong Precision Industry Ltd.

As shown in Figure 1B, the two battery cells (nominal capacity: 40 Ah), were donated by Contemporary Amperex Technology Co., Ltd. These were used for all battery cooling experiments. The detailed properties of the battery cell were listed in Table S3 (Supporting Information). The battery charging/discharging setup can provide 100 A maximum charging/discharging current. The setup was procured from Youpaisi electronics Co., Ltd. (model: YPSDZ‐0550‐4). Thermocouples used in the thermal experiments were unsheathed and procured from Omega Co., LTD. They were calibrated to an uncertainty of ± 0.1 °C. A temperature data recorder Agilent 34970A from Keysight Co., LTD was used for data acquisition and storage. The temperature sampling rate was 1 Hz.

Please refer to Section S14 (Supporting Information) for a description of the devices used for characterization purposes.

### Experimental Methods

In each experimental run, all groups experienced the same discharge‐charge‐discharge‐charge processes to ensure a fair comparison. Initially, battery cells in each group were fully charged. All three groups experienced passive thermal management where these battery cells resided on a thermal insulation layer on an experimental bench. The passive cooling scheme simulates a vehicle stall or unpowered state condition. For each experimental group (A, B, C), six operating conditions with two environmental temperatures (20 and 30 °C) and three charging/discharging currents (50 A, 80 A, and 100 A) were conducted. The 50 A, 80 A, and 100 A current represent 1.25 C, 2.00 C, and 2.50 C, respectively, as the normal capacity of the adopted LIB is 40 Ah. In each operating condition, during the transition of discharging–charging or charging–discharging, the tested batteries of each group underwent an idle period of one minute. Three thermocouples were utilized to measure the temperatures at three locations on the batteries.

## Conflict of Interest

The authors declare no conflict of interest.

## Author Contributions

N.M., Y.M., and J.‐X.W. conceived this project, wrote and revised the manuscript. J.‐X.W. and Y.M. designed the experiments, synthesized the materials, and performed the experiments. J.‐X.W. and N.M. analyzed the results. The project was planned, directed and supervised by N.M. and Y.M. All authors have given approval to the final version of the manuscript.

## Supporting information

Supporting Information

Supplemental Movie 1

Supplemental Movie 2

## Data Availability

The data that support the findings of this study are available from the corresponding author upon reasonable request.

## References

[1] Y. Zhao , Y. Wen , F. Wang , W. Tu , S. Zhang , Y. Wu , J. Hao , Appl. Energ. 2023, 342, 121102.

[2] K. Qin , K. Holguin , J. Huang , M. Mohammadiroudbari , F. Chen , Z. Yang , G.‐L. Xu , C. Luo , Adv. Sci. 2022, 9, 2106116. 10.1002/advs.202106116 PMC9731705 36316243

[3] C. Zhang , Y. Zhang , Z. Nie , C. Wu , T. Gao , N. Yang , Y. Yu , Y. Cui , Y. Gao , W. Liu , Adv. Sci. 2023, 10, 2300506. 10.1002/advs.202300506 PMC10288226 37085926

[4] Q. Liu , T. Meng , L. Yu , S. Guo , Y. Hu , Z. Liu , X. Hu , Small Methods 2022, 6, 2200380. 10.1002/smtd.202200380 35652156

[5] M. Li , G. Cai , J. Holoubek , K. Yu , H. Liu , S. Sarwar , Q. Yan , H. Gao , D. Zhang , H. Zhou , P. P. Mukherjee , S. Lee , B. Jung , Z. Chen , Nano Energy 2022, 103, 107726.

[6] S. Zhang , S. Li , X. Wang , C. Li , Y. Liu , H. Cheng , S. Mao , Q. Wu , Z. Shen , J. Mao , H. Pan , Y. Lu , Nano Energy 2023, 114, 108639.

[7] H. Wang , Q. Wang , C. Jin , C. Xu , Y. Zhao , Y. Li , C. Zhong , X. Feng , J. Harzard. Mater. 2023, 458, 131646. 10.1016/j.jhazmat.2023.131646 37331058

[8] L. Zhang , Q. Duan , X. Meng , K. Jin , J. Xu , J. Sun , Q. Wang , Energ. Convers. Manage. 2022, 252, 115091.

[9] Y. Song , Y. Cui , B. Li , L. Geng , J. Yan , D. Zhu , P. Zhou , J. Zhou , Z. Yan , Q. Xue , Y. Tang , W. Xing , Nano Energy 2023, 116, 108846.

[10] X. Duan , J. Li , Y. Jia , X. Gao , L. Wang , J. Xu , Adv. Sci. 2023, 10, 2302496. 10.1002/advs.202302496 PMC10582443 37555288

[11] A. García , J. Monsalve , S. Martinez‐Boggio , D. Golke , Energ. Convers. Manage. 2023, 276, 116530.

[12] G. Zhang , X. Wei , X. Wang , S. Chen , J. Zhu , H. Dai , Nano Energy 2024, 126, 109621.

[13] T. Dong , S. Zhang , Z. Ren , L. Huang , G. Xu , T. Liu , S. Wang , G. Cui , Adv. Sci. 2024, 11, 2305753. 10.1002/advs.202305753 PMC10870087 38044323

[14] M. R. Palacín , A. De Guibert , Science 2016, 351, 1253292. 26912708 10.1126/science.1253292

[15] S. Lee , H. Lee , Y. J. Jun , H. Lee , Appl. Energy 2024, 353, 122043.

[16] S. Mansour , A. Jalali , M. Ashjaee , E. Houshfar , Energ. Convers. Manage. 2023, 290, 117200.

[17] J.‐X. Wang , B. Cui , C. Salmean , X. Chen , X. Yan , Y. Mao , S. Yao , Nano Energy 2024, 125, 109560.

[18] A. Masias , J. Marcicki , W. A. Paxton , ACS Energy Lett. 2021, 6, 621.

[19] W. Zeng , C. Ma , S. Hu , S. Li , Y. Zhang , Energ. Convers. Manage. 2023, 292, 117378.

[20] J.‐X. Wang , C. Salmean , J.‐X. Li , C. Lei , J. Li , M. Zhong , B. Qi , Y. Mao , Nano Materials Science 2023, 10.1016/j.nanoms.2023.11.005.

[21] D. G. Atinafu , B. Y. Yun , Y. U. Kim , S. Kim , Small Methods 2023, 7, 2201515. 10.1002/smtd.202201515 36855164

[22] J.‐X. Wang , J. Qian , N. Wang , H. Zhang , X. Cao , F. Liu , G. Hao , Renew. Energ. 2023, 213, 75.

[23] S. Kim , T. Yang , N. Miljkovic , W. P. King , Int. J. Heat Mass Trans. 2023, 213, 124263.

[24] T. Yang , W. P. King , N. Miljkovic , Cell Reports Physical Science 2021, 2, 100540.

[25] T. Yang , P. V. Braun , N. Miljkovic , W. P. King , Int. J. Heat Mass Trans. 2021, 170, 121033.

[26] W. Fu , Y. Gurumukhi , X. Yan , V. S. Garimella , W. P. King , N. Miljkovic , Nat. Energy 2022, 7, 270.

[27] H. Moon , N. Mijkovic , W. P. King , Int. J. Heat Mass Trans. 2020, 153, 119591.

[28] Y. Guo , T. Hou , J. Wang , Y. Yan , W. Li , Y. Ren , S. Yan , Adv. Sci. 2023, 2304580. 10.1002/advs.202304580 PMC11462306 37963852

[29] L. Liu , Y. Zhang , S. Zhang , B. Tang , Adv. Sci. 2023, 10, 2207652. 10.1002/advs.202207652 PMC10401154 37226721

[30] A. NematpourKeshteli , M. Iasiello , G. Langella , N. Bianco , Energ. Convers. Manage. 2023, 291, 117268.

[31] T. Yang , J. G. Kang , P. B. Weisensee , B. Kwon , P. V. Braun , N. Miljkovic , W. P. King , Appl. Phys. Lett. 2020, 116, 071901.

[32] S. Zhang , Z. Li , Y. Yao , L. Tian , Y. Yan , Nano Energy 2022, 100, 107476.

[33] S. Chen , Z. Chen , Z. Hu , S. Yu , J. Zhou , H. Xiang , M. Zhu , Chem. Eng. J. 2023, 476, 146833.

[34] Z. Chai , M. Fang , X. Min , Nano Energy 2024, 124, 109437.

[35] Z. Tang , Y. Gao , P. Cheng , Y. Jiang , J. Xu , X. Chen , A. Li , G. Wang , Nano Energy 2022, 99, 107383.

[36] X. Chen , P. Cheng , Z. Tang , X. Xu , H. Gao , G. Wang , Adv. Sci. 2021, 8, 2001274. 10.1002/advs.202001274 PMC8097397 33977039

[37] Y. He , Y. Dong , Y. Zhang , Y. Li , H. Li , Adv. Sci. 2023, 10, 2207426. 10.1002/advs.202207426 PMC10214271 36950760

[38] G. Sadeghi , M. Mehrali , M. Shahi , G. Brem , A. Mahmoudi , Energ. Convers. Manage. 2022, 269, 116176.

[39] X. Zhang , K. Sun , H. Liu , J. Chen , X. Yan , Y. Kou , Q. Shi , Nano Energy 2024, 121, 109256.

[40] C. Zhong , S. Weng , Z. Wang , C. Zhan , X. Wang , Nano Energy 2023, 117, 108894.

[41] P. K. Tyagi , R. Kumar , Z. Said , Nano Energy 2022, 93, 106834.

[42] Q. Zhu , P. J. Ong , S. Goh , R. Yeo , S. Wang , Z. Liu , X. J. Loh , Nano Materials Science 2024, 6, 115.

[43] J.‐X. Wang , Y.‐Z. Li , S.‐N. Wang , H.‐S. Zhang , X. Ning , W. Guo , Energ. Convers. Manage. 2016, 123, 232.

[44] J. Wang , Y. Li , X. Liu , C. Shen , H. Zhang , K. Xiong , Chinese J. Aeronaut. 2021, 34, 1.

[45] M. A. Gerkman , G. G. D. Han , Joule 2020, 4, 1621.

[46] Y. Mao , M. Zhong , J. X. Wang , Appl. Energ. 2023, 352, 121882.

[47] R. Feng , P. Huang , Z. Tang , Y. He , Z. Bao , Energ., Convers. Manage. 2022, 272, 116359.

[48] W.‐Y. Chen , X.‐L. Shi , J. Zou , Z.‐G. Chen , Small Methods 2022, 6, 2101235.

[49] D. Karimi , H. Behi , M. Berecibar , J. V. Mierlo , Appl. Energ. 2023, 339, 120987.

[50] J. Lin , H. N. Chu , K. Thu , M. Wojtala , F. Gao , K. J. Chua , Energ. Convers. Manage. 2023, 283, 116948.

[51] Q. Zhang , S. Wang , X. Wang , Y. Jiang , J. Li , W. Xu , B. Zhu , J. Zhu , Small Methods 2022, 6, 2101379. 10.1002/smtd.202101379 35212488

[52] S. Bae , M. Kim , G. Kang , H. S. Lee , I. S. Kim , S. W. Choi , J. G. Kang , Chem. Eng. J. 2024, 483, 149245.

[53] Q. Yan , F. E. Alam , J. Gao , W. Dai , X. Tan , L. Lv , J. Wang , H. Zhang , D. Chen , K. Nishimura , L. Wang , J. Yu , J. Lu , R. Sun , R. Xiang , S. Maruyama , H. Zhang , S. Wu , N. Jiang , C.‐T. Lin , Adv. Funct. Mater. 2021, 31, 2104062.

[54] J.‐X. Wang , H. Lai , M. Zhong , X. Liu , Y. Chen , S. Yao , Small Methods 2023, 7, 2300139. 10.1002/smtd.202300139 37129546

[55] X. K. Yu , Y. B. Tao , Int. J. Heat Mass Trans. 2022, 198, 123433.

[56] J. Cao , Z. Ling , S. Lin , Y. He , X. Fang , Z. Zhang , Chem. Eng. J. 2022, 433, 133536.

[57] Y. Li , C. Lin , J. Huang , C. Chi , B. Huang , Glob. Chall. 2021, 5, 2000058. 33437525 10.1002/gch2.202000058 PMC7788633

[58] Y. Lu , X. Cheng , G. Tian , H. Zhao , L. He , J. Hu , S. M. Wu , Y. Dong , G. G. Chang , S. Lenaerts , S. Siffert , G. V. Tendeloo , Z.‐F. Li , L.‐L. Xu , X.‐Y. Yang , B.‐L. Su , Nano Energy 2018, 47, 8.

[59] Y. Dong , J. Li , X. Y. Yang , Sci. Bull. 2022, 67, 1943. 10.1016/j.scib.2022.09.006 36546200

[60] F. Wang , X. Liao , H. Wang , Y. Zhao , J. Mao , D. G. Truhlar , Interdiscip. Mater. 2022, 1, 517.

[61] K. Wang , W. Wang , Y. Wang , M. Wang , Chem. Eng. J. 2024, 481, 148538.

[62] S. Singh , M. Jennings , S. Katragadda , J. Che , N. Miljkovic , Appl. Therm. Eng. 2023, 232, 120990.

[63] S. Singh , M. Olyaei , K. Jiang , Y. Gurumukhi , K. Goodson , M. Asheghi , N. Miljkovic , in ASME 2023 International Electronic Packaging Technical Conference and Exhibition, San Diego, Californi, USA 2023.

[64] A. J. Mahvi , K. Boyina , A. Musser , S. Elbel , N. Miljkovic , Int. J. Heat Mass Trans. 2021, 172, 121162.

[65] J.‐X. Wang , J. Qian , J.‐X. Li , X. Wang , C. Lei , S. Li , J. Li , M. Zhong , Y. Mao , J. Colloid Inter. Sci. 2024, 658, 748. 10.1016/j.jcis.2023.12.095 38142625

[66] J.‐X. Wang , Z. Wu , M.‐L. Zhong , S. Yao , Int. Commun. Heat Mass 2021, 126, 105387.

[67] F. Liu , J. Xu , S. Yan , Y. Zhou , Y. Zhang , Chem. Eng. J. 2024, 493, 152737.

[68] J.‐X. Wang , Y.‐Z. Li , G.‐C. Li , K. Xiong , X. Ning , Int. J. Heat Mass Trans. 2017, 114, 715.

[69] Z. Teng , M. Zhong , Y. Mao , E. Li , M. Guo , J. X. Wang , Energ. Convers. Manage. 2022, 261, 115524.

[70] J.‐X. Wang , M. Zhong , Z. Wu , M. Guo , X. Liang , B. Qi , Appl. Energ. 2022, 322, 119517.

[71] X. Liu , R. Chen , F. Li , D. Zhou , J. Zou , IEEE Trans. Power Electron. 2024, 39, 10605.

[72] X. Liu , F. Gao , H. Niu , G. Sun , T. Wang , H. Wang , IEEE Trans. Power Electron. 2024, 39, 1749.

[73] M. Chen , S. Chen , M. Liao , M. Jin , Y. Zhao , G. Zhou , L. Shui , Z. Yan , Energ. Convers. Manage. 2022, 266, 115808.

[74] X. Wang , M. Gao , Y. M. Lee , M. Salla , F. Zhang , S. Huang , Q. Wang , Nano Energy 2022, 93, 106864.

[75] Y.‐A. M. Xi , Y.‐Z. Li , Y.‐H. Chen , H.‐H. Jiang , Z.‐B. Huang , Appl. Energ. 2024, 371, 123743.

[76] Y. Zhang , J. Feng , J. Qin , Y. L. Zhong , S. Zhang , H. Wang , J. Bell , Z. Guo , P. Song , Adv. Sci. 2023, 10, 2301056. 10.1002/advs.202301056 PMC10460903 37334882

[77] Y. Li , T. Wang , X. Li , G. Zhang , K. Chen , W. Yang , Appl. Energ. 2022, 327, 120109.

