# Supplementary material for: Nano‐Enhanced Graphite/Phase Change Material/Graphene Composite for Sustainable and Efficient Passive Thermal Management
Source: Adv Sci (Weinh). 2024 Aug 9;11(38):2402190. doi: 10.1002/advs.202402190 (PMC11481206; doi:10.1002/advs.202402190)
Supplement: Supplementary file 1 — Supporting Information [file ADVS-11-2402190-s001.docx]

Supporting Information

Nano-Enhanced Graphite/Phase Change Material/Graphene Composite for Efficient Passive Thermal Management

Ji-Xiang Wang, Yufeng Mao,* Nenad Miljkovic*

This file includes:

Section S1: Expanded graphene/PCM Composite Fabrication Processes

Section S2: Thermal Conductivity Measurement

Section S3: Differential Scanning Calorimetry (DSC) Results

Section S4: Graphene Solution Preparation

Section S5: Graphene Coating Process

Section S6: Further Characterization of the Graphene Coating

Section S7: Thermogravimetric Analysis (TGA) of the Composite

Section S8: Properties of the Utilized Battery Cells

Section S9: Characterization of the Original Battery Surface

Section S10: Photograph of the Group C Experimental Battery Prototype

Section S11: Properties of Components Used in the Active Cooling Scheme

Section S12: Durability Experiments of the EG/PCM/graphene Composite

Section S13: Flammability Test of the EG/PCM/graphene Composite

Section S14: Characterization Instruments and Methods

Other Supporting Information for this manuscript include the following:

Movie S1. Infrared view of the whole experimental process on Group D (charging/discharging current: 2.50 C; environmental temperature: 30°C)

Movie S2. Flammability test

**S1. Expanded graphene/PCM Composite Fabrication Processes**

| 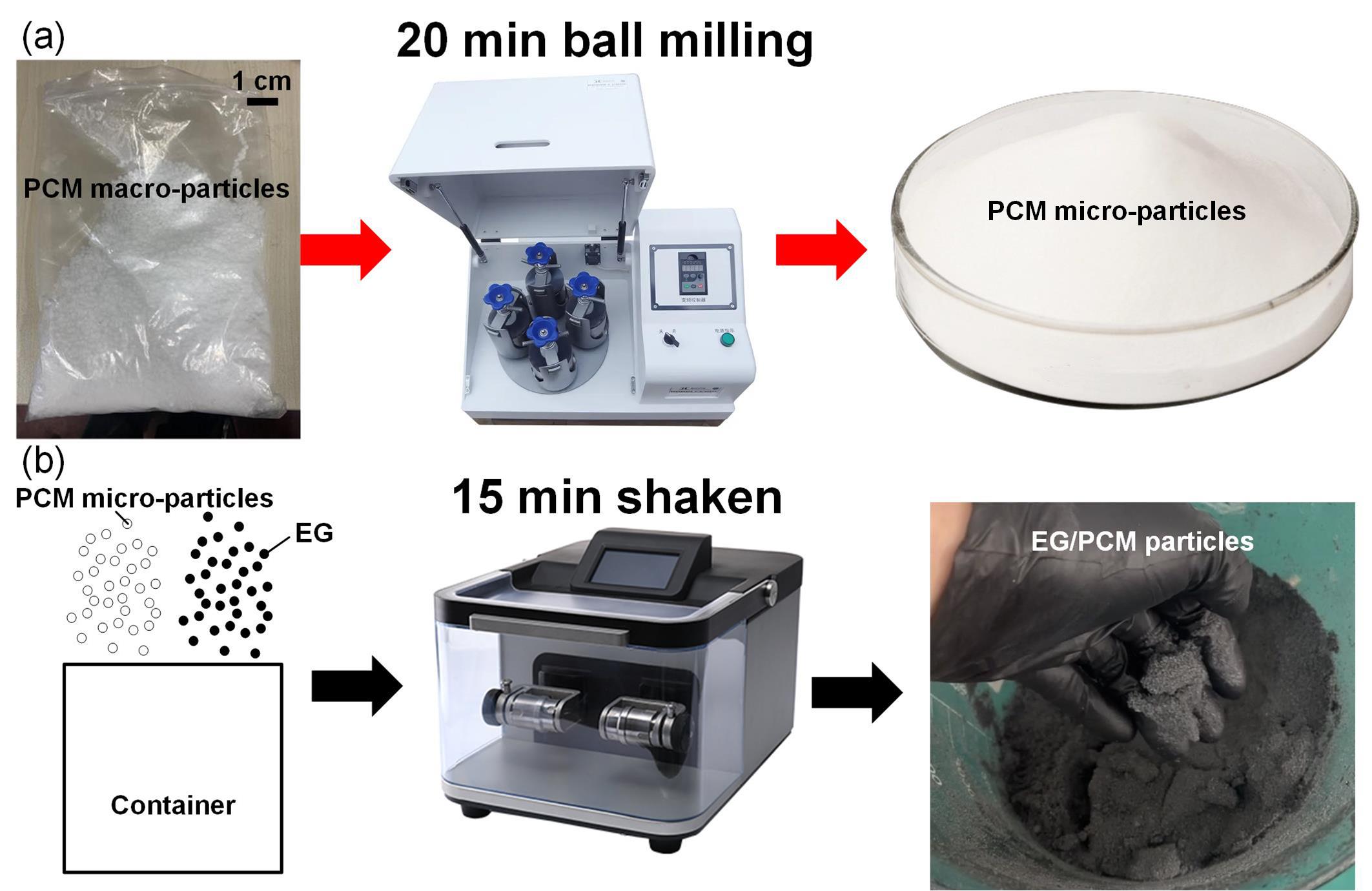  **Figure S1. Procedures to fabricate the EG/PCM particles.** (a) Procedures to fabricate paraffin micro particles. (b) Procedures to fabricate EG/PCM particles. |
| --- |

This section details the procedures used to obtain the desired EG/PCM composite. Figure S1(a) presents the original appearance of the pristine paraffin wax, which was in the form of macro-particles having diameters of 2 ~ 3 mm. First, a certain mass of the paraffin wax macro-particles underwent a 20 min low-energy ball milling step to obtain paraffin micro-particles as shown in the right-side of Figure S1(a). As shown in Figure S1(a), we put certain masses of paraffin macro-particles and expanded graphene (EG) into a container, which then, underwent a 15 min shake step. During the shake, the paraffin micro-particles can be coated with EG evenly^[1]^. The mixture was subsequently heated to 60°C for approximately 10 min and shaken again for about 10 min to have the PCM melted and captured by the capillary from the EG’s inside structure. Finally, the incompact EG/PCM particles were filled into a steel mold and compressed into desired composite block, as shown in Figure 2C, at the compression pressure around 18 MPa.

**S2. Thermal Conductivity Measurement**

| 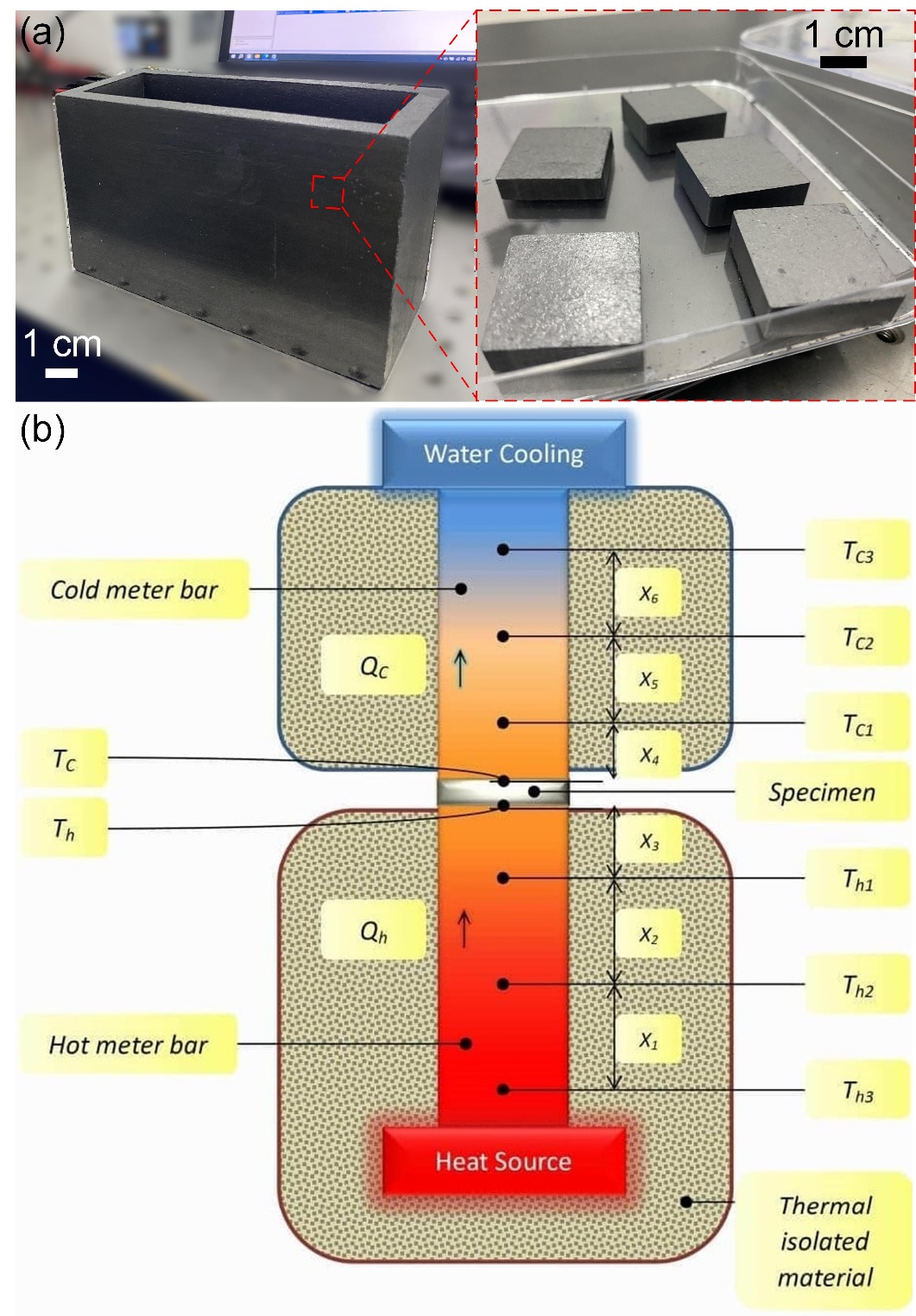  **Figure S2. Thermal conductivity measurement.** (a) Specimen fabrication photographs. (b) Experimental test rig used for thermal conductivity measurement. Schematic not to scale^[2]^. |
| --- |

We used mechanical processing approaches to obtain specimens that were tested. The specimens, shown in Figure S2(a), were directed obtained from the EG/PCM PCC. The width and length of these specimens was approximately 25 mm × 25 mm. The specimen height $h_{s}$ ranged between 1.5 mm to 3.0 mm. Then, these specimens were loaded into an interfacial thermal resistance and thermal conductivity measurement system (model: LW9389) obtained from Longwin Electronic Science & Technology Co., Ltd. The system was used to measure the effective thermal conductivity. Figure S2(b) shows a schematic of the system used for thermal conductivity measurement. The heat flux through the hot calorimeter bar is defined as **$q_{h}=\lambda_{m}\frac{T_{h3}-T_{h1}}{X_{1}+X_{2}}$** and the heat flux through the cold calorimeter bar is **$q_{C}=\lambda_{m}\frac{T_{C3}-T_{C1}}{X_{5}+X_{6}}$** where $\lambda_{m}$ is the thermal conductivity of the calorimeter bar material. The heat flux through the specimen is defined as **$q_{s}=\frac{q_{h}+q_{C}}{2}$**. The hot side interface temperature of the specimen is calculated as **$T_{h}=T_{h1}-\frac{X_{3}}{X_{1}+X_{2}}\left(T_{h3}-T_{h1}\right)$** and the cold side interface temperature is calculated as **$T_{C}=T_{C1}-\frac{X_{4}}{X_{5}+X_{6}}\left(T_{C3}-T_{C1}\right)$**. Therefore, the thermal conductivity of the specimen at the thermal steady state is defined as:

| **$\lambda_{s}=\frac{h_{s}q_{s}}{T_{h}-T_{C}}$**. | (S1) |
| --- | --- |

During measurement, the heat source generated 150 W. At steady state, *T_h_* was well above the melting point (> 53°C) and *T_C_* was below the melting point so the state of the EG/PCM was in a liquid-solid mixed phase when measuring its thermal conductivity.

**S3. Differential Scanning Calorimetry (DSC) Results**

DSC results of the pure paraffin wax and the integrated EG/PCM composite are presented in Figure S3 where the key quantitative results are listed in Table S1.

| 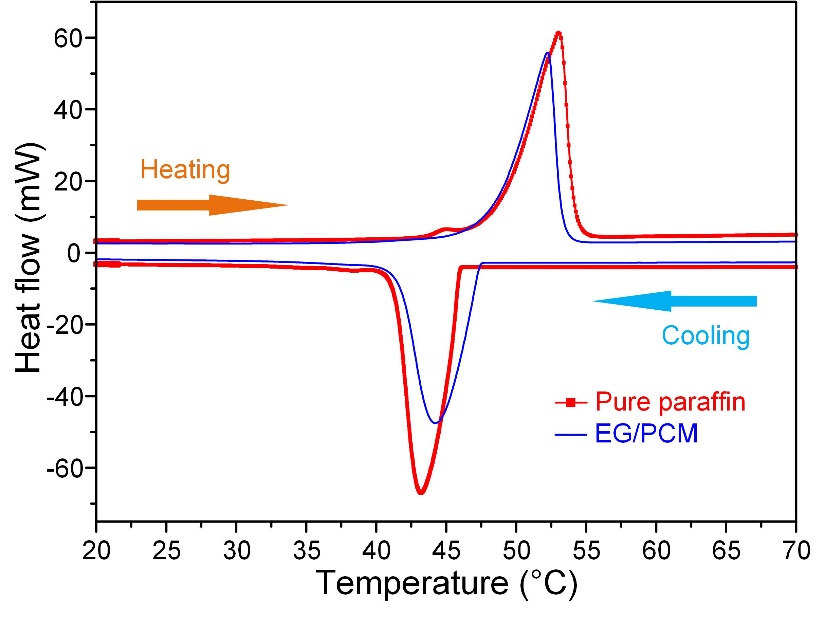  **Figure S3. Differential scanning calorimetric curves of the pure paraffin wax and EG/PCM composite.** |
| --- |

**Table S1.** Key measured parameters of the paraffin and EG/PCM composite using the DSC results.

| Material | Phase-change start point  (°C) | Phase-change end point  (°C) | Latent heat  (J/g) |
| --- | --- | --- | --- |
| Pristine paraffin (heating) | 49.0 | 54.1 | 254.0 |
| Pristine paraffin (cooling) | 45.9 | 41.4 | 277.8 |
| EG/PCM (heating) | 48.3 | 53.2 | 216.1 |
| EG/PCM (cooling) | 47.4 | 41.7 | 213.9 |

**S4. Graphene Solution Preparation**

The procedure used to prepare the graphene solution is as follows: (1) dissolve 60 g of absolute ethyl alcohol in 325 g of deionized water; (2) disperse the prepared graphene into the ethanol solution; (3) dissolve 75 g of waterborne synthetic polyurethane, 15 g of Di (propylene glycol) methyl ether, and 1 g of 1-Methyl-2-pyrrolidinone into the solution. The mass fraction of the graphene is approximately 1.50 wt%. Before coating, the graphene solution should undergo 24 h of ball milling.

**S5. Graphene Coating Process**

One side of the copper foil (CF) was utilized as the substrate for the graphene coating. The substrate was first immersed into an ultrasonic bath of acetone for 30 min, then rinsed with deionized water, and dried with a nitrogen gas flow. Before coating, the substrate was treated by O_2_ plasma (40 W for 2 min) for enhancement of wettability and surface activation. After the plasma treatment, the substrate was put into a vacuum ultrasonic spray machine UAM4000 procured from Cheersonic LTD. equipped with a nozzle with 40 kHz frequency. The graphene solution adopted for spray coating should undergo 24 h of high-energy ball milling prior to spraying. The sprayed surface should be perpendicular to the spray nozzle. The spray coating processes were conducted in an alternating zig-zag pattern with a 5 mm gap to cover the entire surface area with the graphene solution at a flow rate of 0.6 mL/min and air pressure of 20 kPa. The nozzle speed was regulated to be 50 mm/s. The spray coating ran 15 times for each substrate. The nanosheet graphene was spray-deposited and annealed at 110°C for 20 min to remove excess solution and guarantee a strong graphene-substrate bond.

**S6. Further Characterization of the Graphene Coating**

| **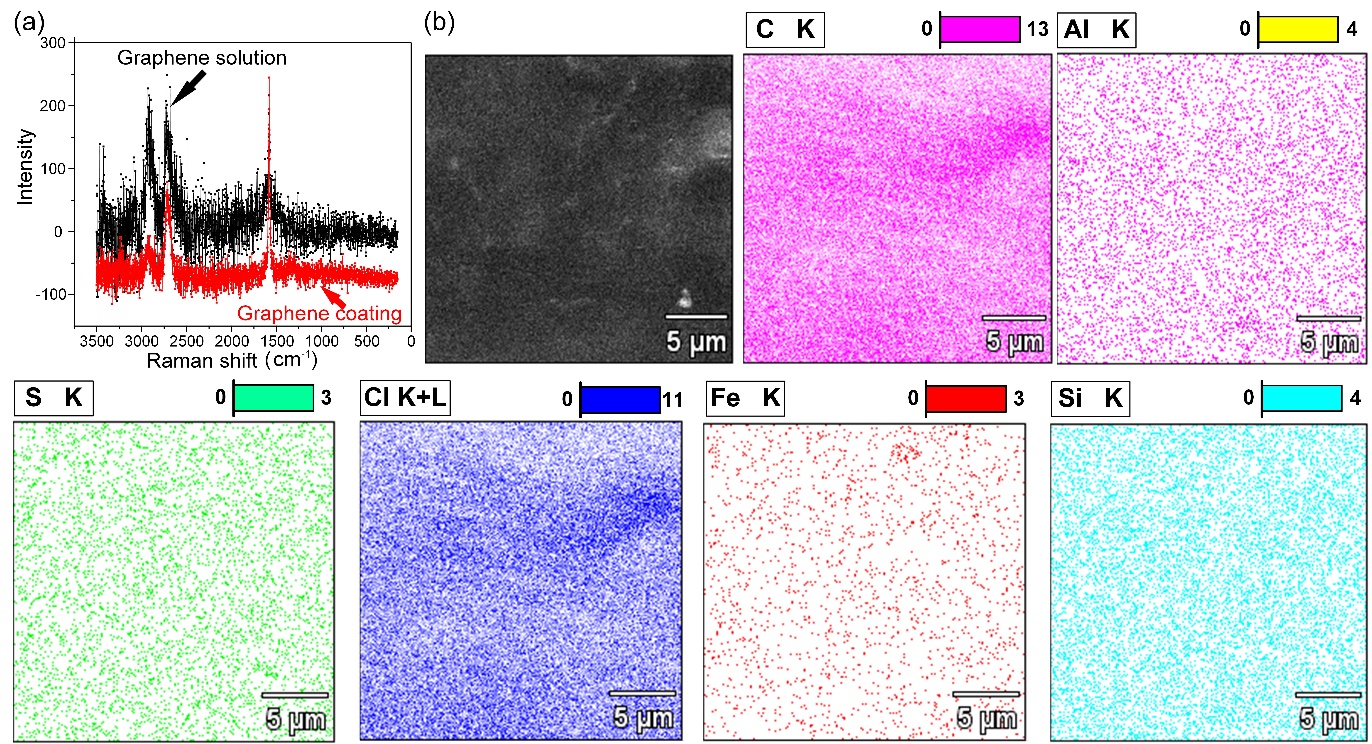**  **Figure S4.** (a) Raman spectra of both the graphene solution and graphene coating. (b) Graphene coating multiple elemental distributions obtained through elemental mapping. |
| --- |

Figure S4(a) shows the Raman spectra of both the prepared graphene solution and graphene coating. The mapping suggests a strong graphene signal. For the graphene solution signal, the peaks at 2923 cm^-1^ and 2713 cm^-1^ are the C-H asymmetric and symmetric stretching vibration spectra, respectively. The G peak is seen at 1581 cm^-1^, reflecting the sp2 hybridization of the C atom. The signal D peak resides at 1304 cm^-1^ where the ratio between $I_{(D)}$ and $I_{(G)}$ is approximately 0.1. This low ratio indicates that the utilized graphene crystal structure is relatively complete with few defects. The graphene coating signal is very similar to the signal obtained from the graphene solution.

Elemental mapping of the black graphene coating yields the images in Figure S4(b), with elemental C having the strongest signal. X-ray fluorescence (XRF) analysis of the graphene coating (Table S2) allows calculation of the mass fraction of each element within the graphene coating.

**Table S2.** Results of the graphene coating composition using X-ray fluorescence.

| Element | C | Si | Cl | Al | Fe | S | Zr | Cr | Bi |
| --- | --- | --- | --- | --- | --- | --- | --- | --- | --- |
| (%) | 99.8873 | 0.0866 | 0.0102 | 0.0051 | 0.0034 | 0.0023 | 0.0022 | 0.0019 | 0.0011 |

Figure S5 displays the X-ray photoelectron spectroscopy (XPS) results of the utilized graphene coating. In Figure S5 (a), the binding energy of 284.8 eV is attributed to the C-C bonds. Oxygen-containing groups such as the C-O-C bond (286.2 eV) and O-C˭O (288.5 eV) show very low intensities. Figure S5 (b), (c), and (d) indicate the signals of Si 2p (102.5 eV), S 2p (169 eV), and Al 2p (75 eV), respectively.

| 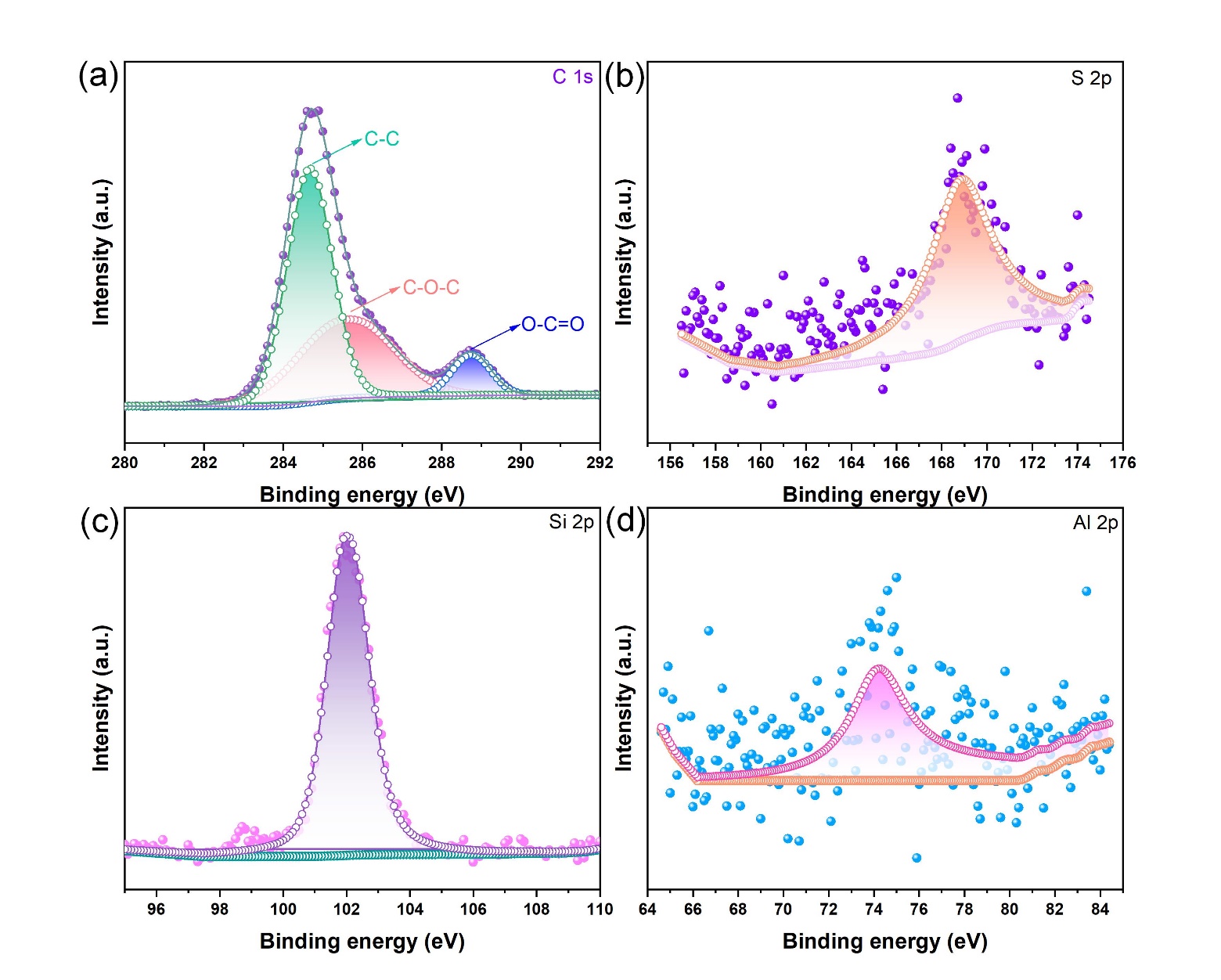  **Figure S5. XPS characterization of the the graphene coating.** |
| --- |

Further morphological characterization of the coated graphene is shown in Figure S6.

| 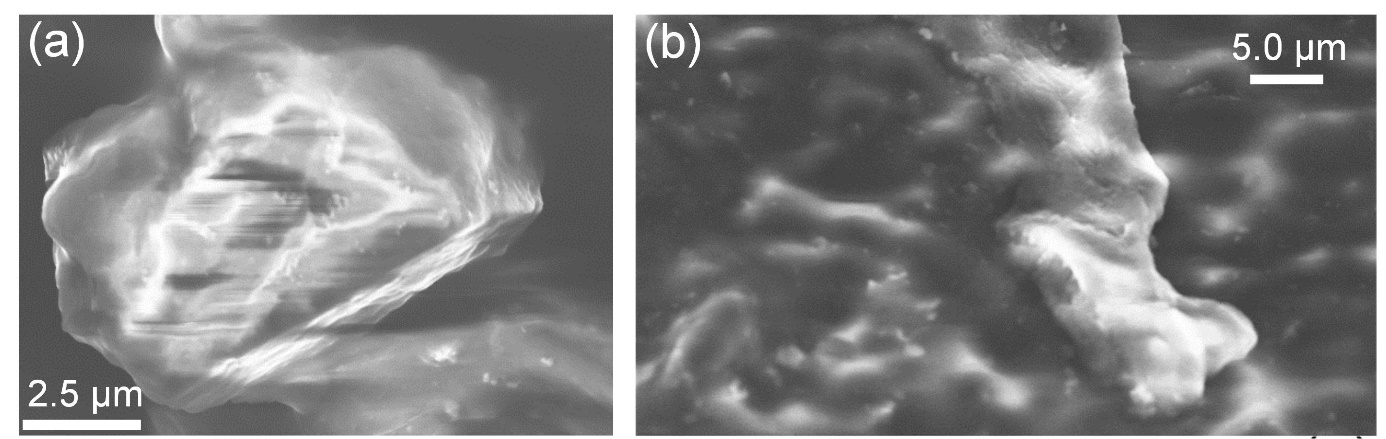  **Figure S6. SEM characterization of the coated graphene.** |
| --- |

**S7. Thermogravimetric Analysis (TGA) of the Composite**

| **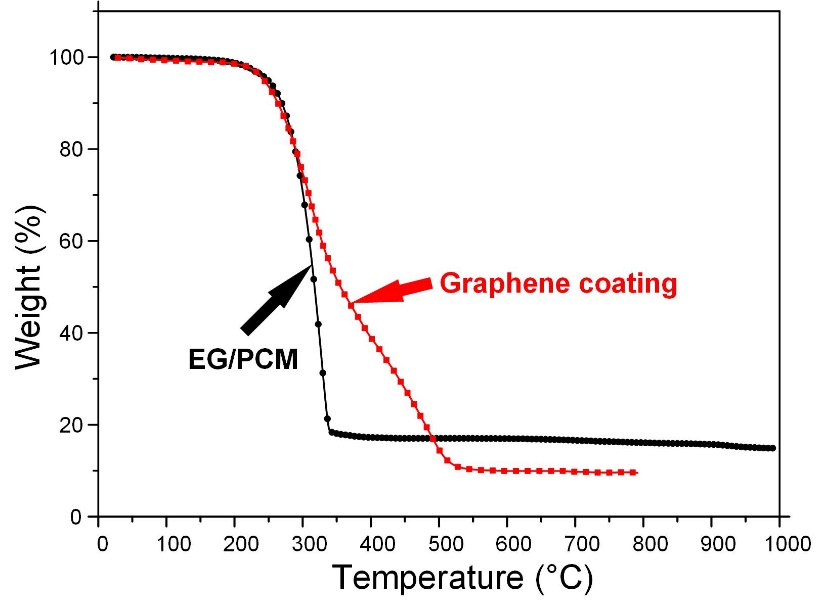**  **Figure S7. TGA of the EG/PCM and graphene coating.** |
| --- |

Figure S7 shows that mass loss for both the EG/PCM and the graphene coating begins at temperatures above 200°C, which is suitable for thermal management of batteries where the operating temperature should never exceed 100°C.

**S8. Properties of the Utilized Battery Cells**

**Table S3.** Specifications of a battery cell adopted in this study.

| Category | Unit | Specification |
| --- | --- | --- |
| Nominal capacity | Ah | 40 |
| Nominal voltage | V | 3.7 |
| Standard discharging current | A | 13.33 (1/3C-rate) |
| Weight | g | 795 |
| Geometric dimensions | mm | 73.7 × 27.2 × 100 (length × width × height) |

**S9. Characterization of the Original Battery Surface**

| 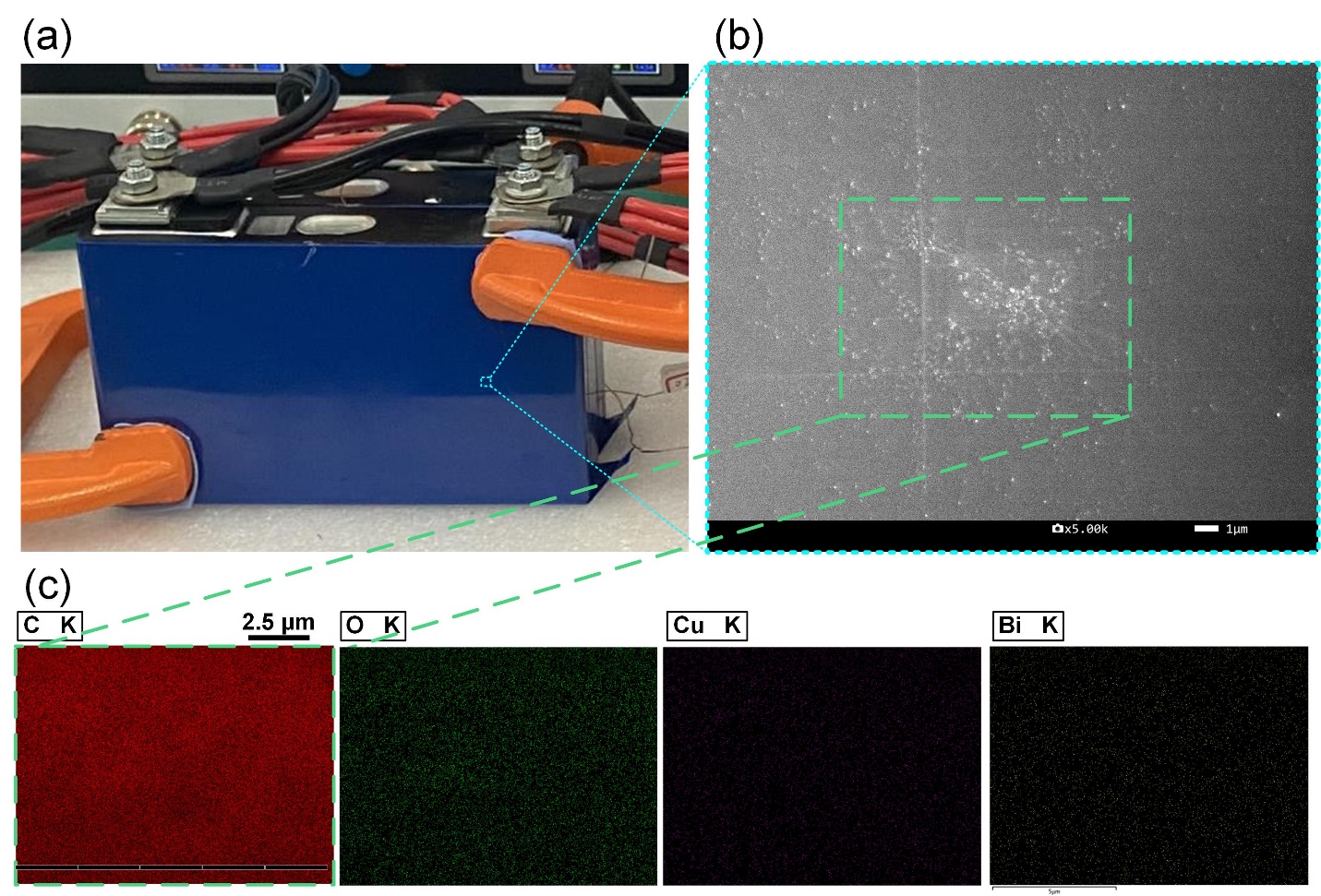  **Figure S8. Original battery surface characterization.** (a) Digital image of the original battery. (b) Surface characterization of the original battery surface. (c) Elemental mapping images of the original battery surface. |
| --- |

Characterization of the original blue battery surface is presented in Figure S8. Figure S8(b) shows an SEM image of the battery surface. Elemental mapping of the surface yielded the images shown in Figure S8(c), with elemental carbon having the strongest signal. X-ray fluorescence analysis of the blue battery surface, listed in Table S4, allows calculation of the mass fraction of each element of the surface. The characterization shows that the original surface of the battery is a common blue paint.

**Table S4.** XRF results of the blue battery surface.

| Element | C | O | Cu | Bi |
| --- | --- | --- | --- | --- |
| Concentration (%) | 99.8873 | 0.0866 | 0.0102 | 0.0011 |

**S10. Photograph of the Group C Experimental Battery Prototype**

| 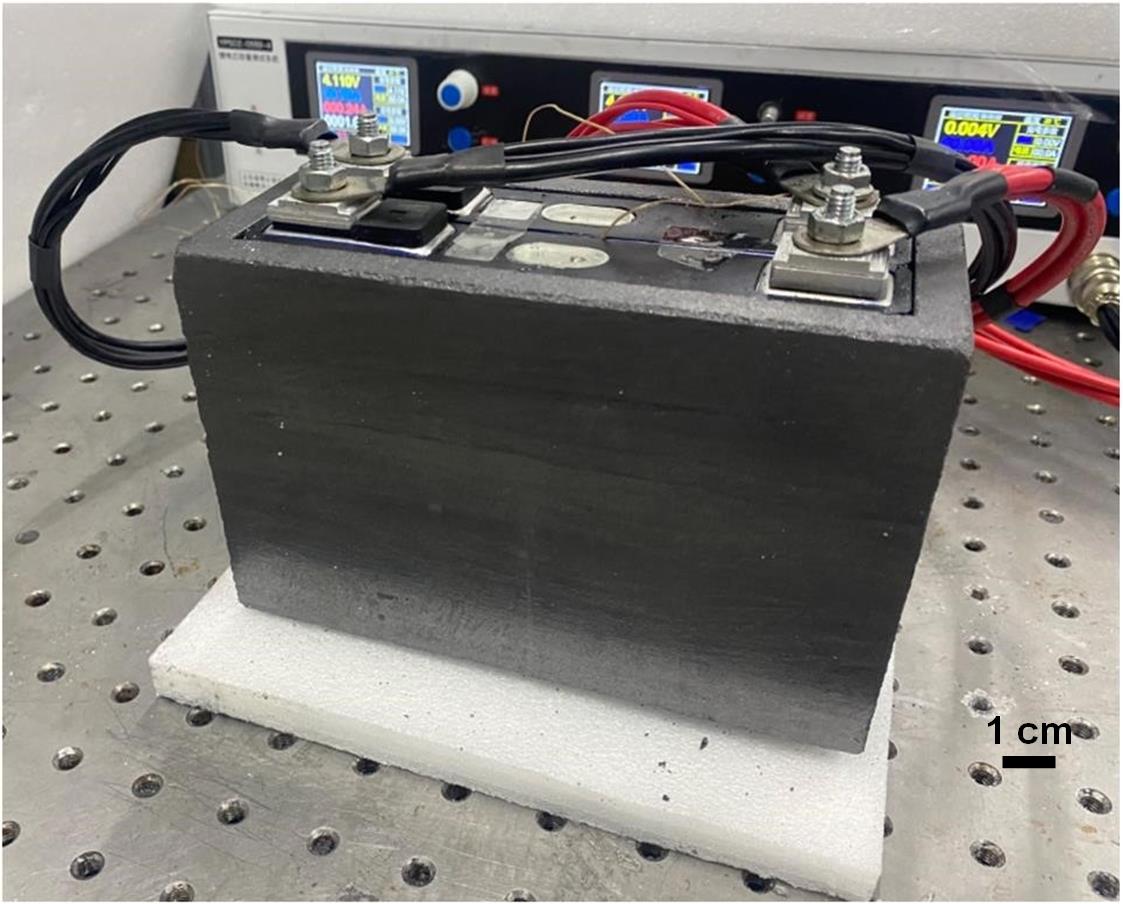  **Figure S9. Photograph of the Group C prototype without the graphene coating.** |
| --- |

**S11. Properties of Components Used in the Active Cooling Scheme**

The heat pipe length × width × thickness is 400 mm × 40 mm × 3 mm. As shown in the inset of Figure 6A, the internal structure of the heat pipe has sawtooth-like fins, which can enhance the heat transfer. The fluid in the heat pipe is an n-pentane (50 wt%)-acetone (50 wt%) binary mixture. Prior to filling the fluid, the heat pipe was first vacuumed. The fill ratio was 50%. The heat pipe working temperature range is approximately -10°C to 150°C. The total weight of the heat pipe was 72.2 g. In addition to the heat pipe, the two aluminum fin sets having a total weight of 538 g were added to the air-cooled condenser region.

**S12. Durability Experiments of the EG/PCM/graphene Composite**

| 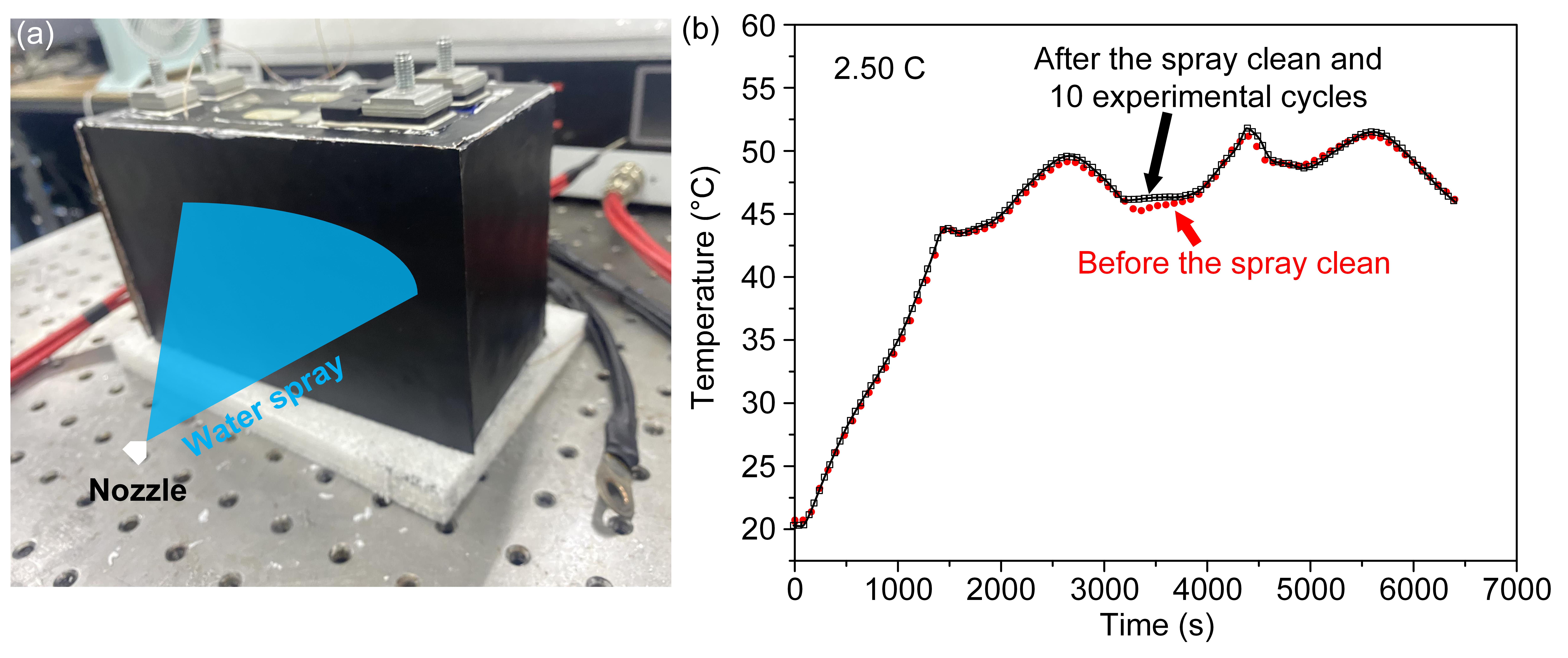 **Figure S10. Durability test of the EG/PCM/graphene composite (Group D).** (a) Photograph with overlaid schematic showing the spray cleaning of Group D. (b) Thermal results (2.50C at ambient temperature of 20°C) before and after spray cleaning. In the experimental design, the spray cleaning process serves as an analogous representation of the typical vehicle cleaning procedure. |
| --- |

This section provides a detailed description of the durability testing protocol and results for the EG/PCM/graphene composite. The testing procedure involved exposing the sample, denoted as Group D, to open-air conditions for a duration of two months. Following this period, a water spray was generated to remove the accumulated dust using a spray nozzle, the parameters of which are specified in Table S5, for a duration of 1 minute. The water flow rate for the spray was also controlled and is listed in Table S5. Then, Group D was dried by a hairdryer. The cleaning process was repeated two times, after which Group D underwent thermal testing at a charging/discharging current of 2.50 C and an ambient temperature of 20°C. A comparison of the temperature data obtained before and after the spray cleaning process is presented in Figure S10(b). Between these two experiments, 10 more experiments with different charging/discharging currents and experimental temperatures were conducted. The identical nature of the two temperature curves demonstrates high durability of the graphene coating.

**Table S5.** Parameters of the spray nozzle and the operating condition.

| Producer | Type | Orifice diameter (mm) | Spray cone angle (°) | Volumetric flow rate  (L/h) |
| --- | --- | --- | --- | --- |
| Spraying System Co. | pressure-swirl, full-cone | 0.50 | 61.0 | 5 |

**S13. Flammability Test of the EG/PCM/graphene Composite**

| 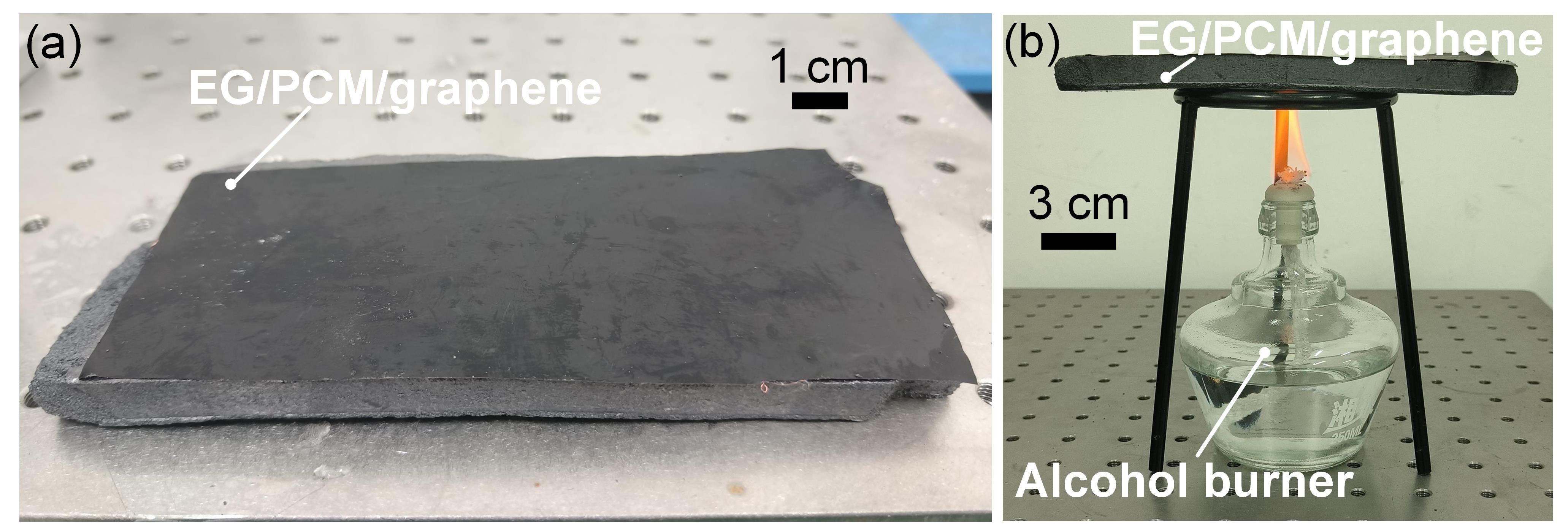 **Figure S11. Flammability test of the EG/PCM/graphene composite.** (a) The photographic view of the composite utilized for the test. (b) The photographic view of the practical flammability test. |
| --- |

Figure S11 shows the flammability test conditions. Figure S11(a) displays the utilized piece of EG/PCM/graphene composite (which weighs 60.8 g). Figure S11(b) shows the flammability test with an alcohol burner. The alcohol burner flame directly contacts the EG/PCM surface, which can simulate the actual condition of a battery fire situation. Video S12 demonstrates that this utilized composite remained unignited for 12 minutes, showcasing its good flame retardancy. This experiment lasted for 12 minutes, a duration sufficient for drivers and passengers to safely evacuate to a secure area in the event of an automobile battery fire.

**S14. Characterization Instruments and Methods**

SEM images and element mapping were attained by a thermal field scanning electron microscope (model: GeminiSEM 300) procured from Carl Zeiss AG. The phase change temperature points and latent heats were acquired by a differential scanning calorimeter (DSC, model: Pyris1 DSC) procured from Perkin-Elmer Inc. with a heating/cooling rate of 10°C/min. Thermal stability of the EG/PCM particles and graphene coating was obtained by a thermogravimetric analyzer (TGA, model: STA 449F3) procured from NETZSCH. The Raman spectra of the graphene coating was attained by a Laser Microscopic confocal Raman spectrometer (model: Renishaw inVia) procured from Renishaw LTD. l MIR reflectance spectra of the graphene coating was measured by an ATR-FTIR Spectrometer (model: Thermo Fisher Scientific Nicolet iS50). A standard reference (a thin gold film) was used. All reflectance (R) spectra measurements were conducted at room temperature (23°C). The spectral emissivity was directly calculated by 1–R.^[3]^ XPS characterizations were obtained using an EscaLab 250Xi XPS manufactured by Thermo Scientific.

**Supplementary References**

[1] T. Li, M. Wu, S. Wu, S. Xiang, J. Xu, J. Chao, T. Yan, T. Deng, R. Wang, Highly conductive phase change composites enabled by vertically-aligned reticulated graphite nanoplatelets for high-temperature solar photo/electro-thermal energy conversion, harvesting and storage. Nano Energy 2021, 89, 106338.

[2] ASTM D5470 Based Thermal Interface Material (TIM) Tests LW-9389 TIM Thermal Interface Material Tester. https://longwinusa.com/services/thermal-interface-material-test/

[3] Y. Li, C. Lin, Z. Wu, Z. Chen, C. Chi, et al., Solution-Processed All-Ceramic Plasmonic Metamaterials for Efficient Solar–Thermal Conversion over 100–727 °C. Adv. Mater. 2021, 33, 2005074.
